# Simultaneous Robot-assisted Laparoscopic Excision of Pre-pyloric Gastrointestinal Stromal Tumor and Cholecystectomy

**DOI:** 10.7759/cureus.1306

**Published:** 2017-06-03

**Authors:** Ahsan Zil-E-Ali, Salman Assad, Furrukh Jabbar

**Affiliations:** 1 General Surgery, Pak Red Crescent Hospital; 2 Department of Medicine, Shifa International Hospital, Islamabad, Pakistan; 3 Department of General, Colorectal & Hepato Pancreatico Biliary Surgery, Appalachian Regional Healthcare, Hazard, Kentucky, Us.

**Keywords:** general surgery, laparoscopic cholecystectomy, gastrointestinal stromal tumor, oncology, minimally invasive technique

## Abstract

Operating on more than a single procedure in a same surgical intervention saves time, anesthesia duration and can increase the overall quality of life by lowering the duration of hospital stay and minimizing patient anxiety. But such interventions require expertise, high surgical performance, and precision in anatomical manipulation. We present a case of an outstanding performance of a unique minimally invasive simultaneous approach of removing a pre-pyloric gastrointestinal stromal tumor (GIST) along with a cholecystectomy by a robot-assisted laparascopic surgical system.

So far, only 33 cases of GIST have been reported in literature that were managed by robot-assistance, and this case is the first of its kind. This is the case of a 60-year-old overweight female who presented for a follow-up for chest discomfort, shortness of breath, chronic gastric reflux and classical features of cholecystitis along with diarrheal and constipation episodes. A gastroduodenoscopy showed a mass in the pre-pyloric area that extended in the luminal cavity. A robot-assisted laparascopic approach was planned, and with precision and surgical expertise the mass was removed along with a cholecystectomy. The surgical specimen were confirmed on histopathology.

## Introduction

Gastrointestinal stromal tumor is a kind of smooth muscle cancer that is a mutation in the cells of Cajal. Theoretically, it can arise from anywhere in the gastrointestinal tract, although there are some major sites known. This tumor can present with many gastrointestinal (GI) symptoms, including GI bleeding, nausea, and pain. The tumor is diagnosed by histopathology, once the mass is removed and confirmed by immunoblotting. If not managed, the prognosis has been known to be poor and follow-up is mandatory to rule out possibilities of metastasis or recurrence. For removing this mass, an immense surgical precision is required.

We present a case of robot-assisted laparascopic approach for excising the tumor in a female patient and simultaneously removing the gall bladder as well. This case depicts the state-of-the-art surgical expertise and procedural success in surgical sciences.

## Case presentation

A 60-year-old female patient presented in the clinic for a follow-up for chest discomfort, which was progressively increasing with shortness of breath and a chronic gastric reflux. Her vitals were within normal limits (pulse: 67, temperature: 97.6 F, blood pressure (BP): 117/86, respiratory rate (RR): 20) and she had a body mass index (BMI) of 29.52 kg/m2 (overweight category). The patient had a massive right upper quadrant (RUQ) pain with classical signs of tenderness, two episodes of vomiting, and a pain that radiated to the right scapula. Her past medical history included multiple episodes of gastric regurgitation with previous cardiovascular intervention for coronary stenting. The patient had a history of diarrheal and constipation episodes for which she had been under treatment with a gastroenterologist. Her gastrointestinal upset was relieved after a four-month long medical treatment along with changes in eating habits. The family history was non-significant. There were no known allergies. The patient reported consuming red wine occasionally and was a recreational smoker.

A routine abdominal ultrasound was done for the cholecystitis, which came positive for gallstones. Considering chronic reflux and chest discomfort with normal troponin and creatine kinase-MB (CK-MB) levels, a gastroduodenoscopy was done to evaluate the possibility of changes in the esophageal epithelium and the angle of His in the cardiac end of the stomach. On visualizing, the epithelium was apparently normal, with normal stomach rugae, color, and texture, and with the absence of a major ulceration. In the distal end of the stomach a mass-like lesion was found in the pre-pylorus that extended in the luminal cavity. The mass was approximately 5-6 cm in length and 7-8 cm in width. It was initially considered that the biopsy of the mass is not a favored procedure, as the extent of blood flow through it was unknown. The patient was informed about the various differentials of the lesion and a robot-assisted laparoscopic intervention was suggested and also a cholecystectomy for the known cholecystitis symptoms.

**Figure 1:**
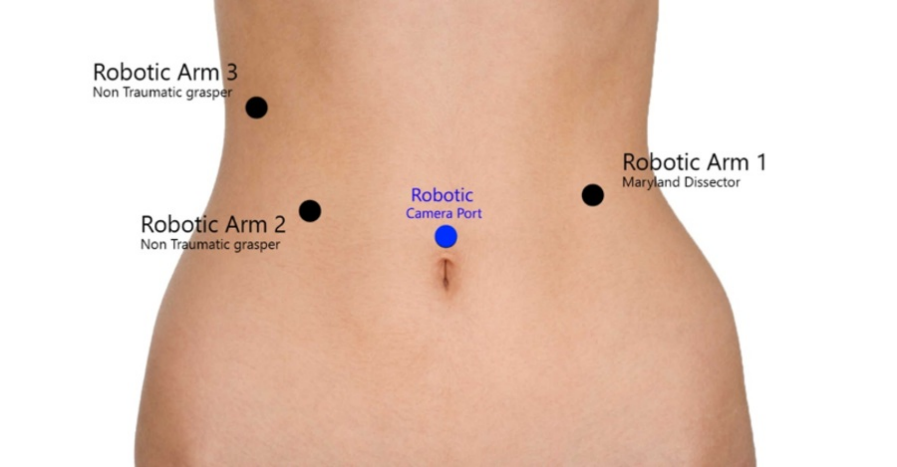
An illustration for the port placement of robotic surgery. Robotic Arm 1, Maryland Dissector in the left mid abdominal region. Robotic Arms 2 and 3, non traumatic graspers on the right side as shown in the image. All three arms are of 8 mm trocars. Robotic camera through 8 mm trocar in the supraumbilical site.

The surgical intervention was scheduled on a da Vinci Surgical Robot System (Intuitive Surgical, CA, USA). Keeping in mind the overweight nature of the patient, appropriate trocars and more accessible port sites were selected. A 5 mm incision was made on the left side of the abdomen and safe intraabdominal access was obtained via opti-view trocar, followed by its replacement with an 8 mm robotic trocar (Robotic Arm 1, Maryland dissector) (Intuitive Surgical, CA, USA). Pneumoperitoneum of 15 mmhg was achieved and then three more robotic trocars were planned including a robotic 8 mm camera trocar supraumbilical and two more 8 mm trocars in RUQ (Figure 1).

**Figure 2:**
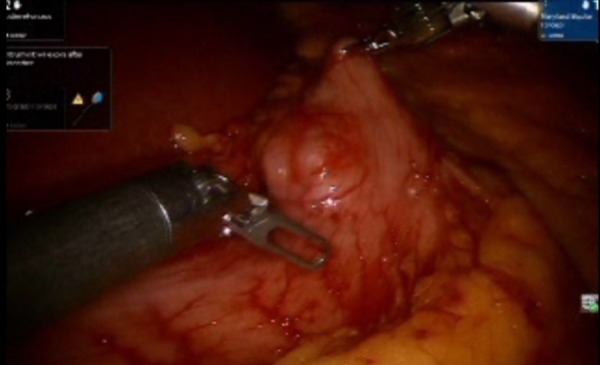
Exploration of the extent of the mass lesion that was later diagnosed as gastrointestinal stromal tumor (GIST). Shown are the robotic arm graspers holding the pre-pyloric area and investigating the extent of the mass.

**Figure 3:**
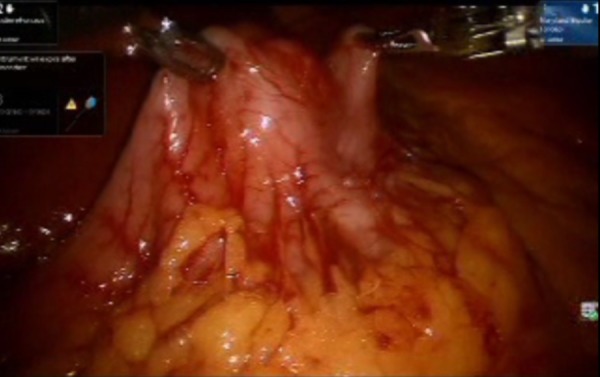
Both robotic arm graspers holding up the lesion in cranio-caudal axis to demarcate the site of excision. The surgeon decided the excision space by evaluating the room available for firing Endo GIA blue load in vertical axis.

**Figure 4:**
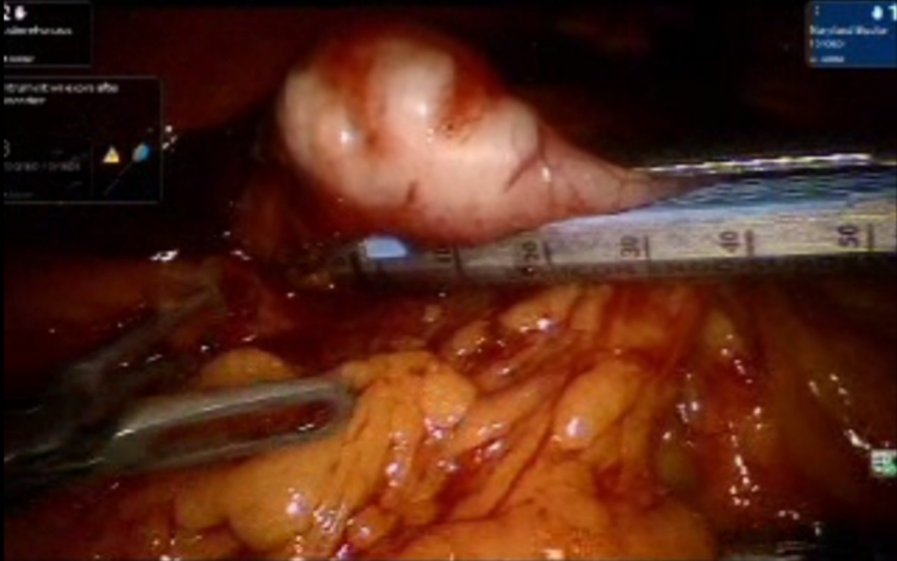
Endo GIA blue load 45 Echelon fired in the vertical axis, separating the mass lesion from the pre-pylorus.

**Figure 5:**
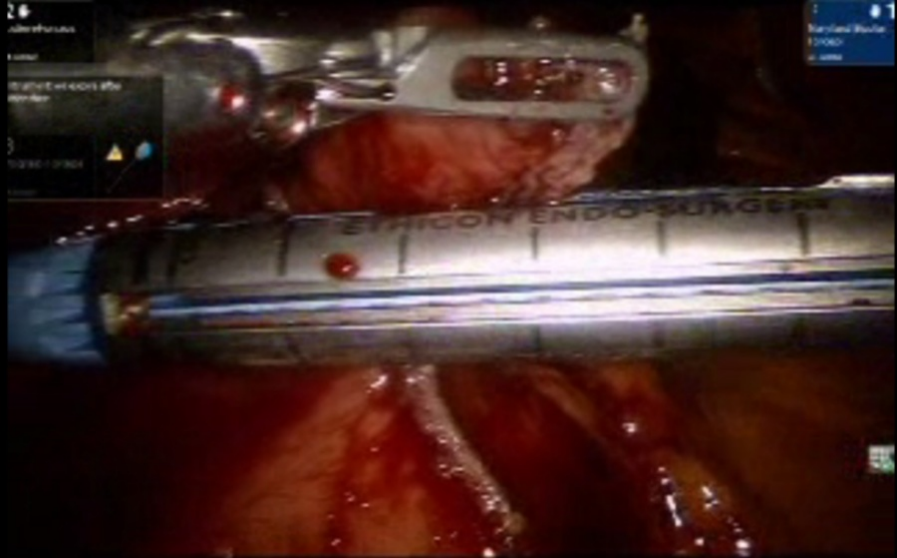
Endo GIA blue load 45 Echelon fired three times in vertical axis separating the lesion and sealing the residual tissue from which the mass is separated. This sealing assures the minimal leakage of luminal contents. A bougie that helps form a bulge is inserted, and this can help as a guide while firing the stapler.

A laparoscopic exploration was performed and a 3 cm mass lesion was identified in the pre-pyloric area. A size 54 bougie was passed through the pyloric channel. The anterior surface of the stomach was held up cranio-caudal with robotic graspers. Endo GIA blue load 45 Echelon (Medtronic, MN, USA) was fired three times in vertical axis to prevent stenosis, and wedge resection of the GIST tumor was successfully performed (Figures 2-5). The specimen was exteriorized with an Endo Catch bag (Medtronic, MN, USA).

**Figure 6:**
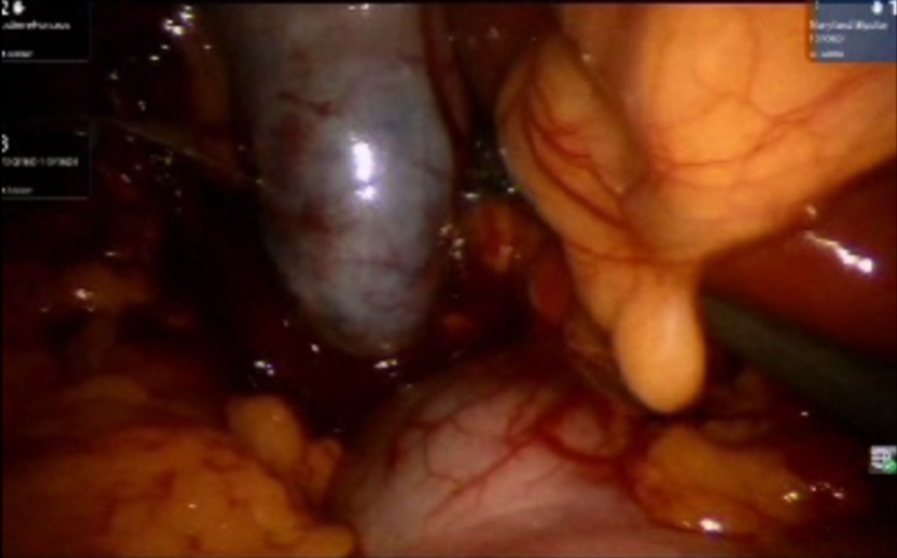
Visualizing the size and extent of the gall bladder prior to its removal. It is assured that no mass lesion is attached to it or the gall bladder is adhered to the liver surface.

**Figure 7:**
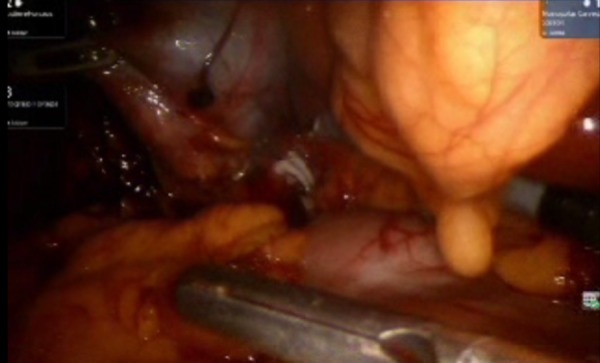
A hallmark step of cholecystectomy. The cystic artery and cystic duct are ligated with clips. This is an essential step for surgeons in ruling out various possibilities of complications.

Using the same trocars and instruments, the liver was retracted and the Calot's triangle was identified (Figure 6). After achieving the critical view of safety, the cystic duct and artery were clipped and divided by an Endo Shears (Medtronic, MN, USA) (Figure 7).

The gallbladder was dissected off its fossa in liver and then retrieved using an Endo Catch bag. The step-by-step intra operative procedure was filmed for academic reference for understanding the simultaneous procedures (Video 1). An esophagogastroduodenoscopy (EGD) was performed to confirm an airtight staple line. A Jackson-Pratt drain (JP drain) was placed in the sub-hepatic fossa to complete the operation. After the surgery, the specimens were sent for pathology; the margins were clear and spindle cell GIST tumor was confirmed. The patient was discharged on second post operative time without complications. The patient was advised to report back in case of symptoms of abdominal tenderness, skin discoloration or pain. Follow-up after six weeks showed no complication. A positron emission tomography-computed tomography (PET-CT) scan was done to look for any malignancy, and none was found.

**Video 1:** This video is a step-by-step guide to remove a mass lesion in the pre-pyloric region along with a cholecystectomy, using the same ports. This technique highlights that no additional ports were inserted, and with expertise, both the procedures were amalgamated into a single intervention. Such surgical procedures can be beneficial for both the patient and the surgical staff.

## Discussion

A literature review was done to evaluate similar cases operated and to compare our prognosis. We found no case of simultaneous excision of GIST and gallbladder by robot-assisted technique or by conventional laparoscopy; although, a total of 33 cases of GIST were managed by robot-assisted technique. There is no similar surgery reported before. It was a cutting-edge approach done with accuracy and precision of the surgical tools used in the robot. Such surgeries can be operated simultaneously with safety. Gastrointestinal stromal tumors (GIST) arise in the smooth muscle pacemaker interstitial cells of Cajal and are usually driven by a mutation in the c-KIT gene (CD 117) that can be observed on immunoblotting. Generally, the tumor presents with GI symptoms that can involve abdominal pain, gastrointestinal bleeding, and trouble swallowing [1]. Intestinal obstruction is rare and the liver is usually the prime site of metastasis. Computed tomography (CT) scanning and endoscopic biopsy are considered as initial diagnostic procedures, following the confirmation on immunoblotting [2]. Excision is the best option to prevent metastasis, but if it is recurrent, then imatinib, a bcr-abl tyrosine kinase inhibitor is used as an adjunct therapy [3]. A close follow-up with early management is key to the treatment.

Robot-assisted surgeries have gained enormous acceptance among surgeons by providing highly magnified three-dimensional imagery that improves hand-eye coordination in real-time. These advanced systems are being used for a number of reconstructive procedures because of their advantages in the range of motion scaling, an extensive optical magnification tool, multidimensional stereoscopic visualization, and an excellent instrument dexterity with tremor filtration that allows delicate motions in small areas in a procedure that would otherwise require advanced laparoscopic expertise [4-5]. This modern way of intervention has addressed many dimensions of surgical expertise and has overcome human error as well; although, research, calibration, and further pilot studies in other fields of abdomino-thoracic surgery will help in making it a more promising instrument.

A study from Italy showed that there were cases of minimal bleeding that were not readily managed in a robotic setting, which contributed to the morbidity, although no fatalities were recorded. It was highlighted that at least 10 solo procedures under the observation of a robotic fellow can shift the learning curve to master it. Still, the huge diameters of the instruments, the limited number of robotic arms, and yet lesser popular academic training are some of the major technical shortcomings of this expensive tool. Further data and evaluation are required for the acceptance of the technology among general surgeons [6]. Our case required a smaller operating field and more precision; hence, robot-assisted intervention was the best option to manage this surgery. Tomulescu, et al. mention in the literature a similar conclusion by investigation that the best indications for robotic surgery are the procedures that require a small operating field, fine precision, and safe intracorporeal sutures [7].

In the past few decades, minimally invasive procedures have reshaped the surgical specialty to an extent that it is difficult for surgeons to come at par with the rapidly expanding nature of technology. But the survival of a surgeon is dependent on learning, adapting, and implementing the expertise in the nature of the current advanced era. In our case report, it becomes evident that treatment for a tumor that was excisable on an open laparotomy with series of post-surgical treatments is totally replaced by a robot-assisted approach and high precision, which is unlikely to be found on a general laparoscopic surgery. The da Vinci Surgical Robot System with its counterparts Al-Khwarzami Surgical System, Zeus Robot, and the most advanced SRI International Robotic Surgeon are evidence of the tremendous efforts of human research, state-of-the-art engineering structure, and a dedication toward dealing with every kind of disease with precision and accuracy. Robotic surgery is tomorrow.

## Conclusions

Robotic-laparascopic approach is a great surgical innovation for obese patients and can assist in manipulating multiple procedures with more precision and accuracy. It is important to note that basic symptoms should not be considered less important as they can be helpful in directing early diagnosis of fatal disease that can assist in prompt treatment. Also, the mass was not biopsied endoscopically, which is a preferred strategy if the extent of the blood flow is not known. Overall, the case helps us understand that GIST may present with very basic enteric symptoms and it requires expertise to approach a certain diagnosis and apply the right tools for patient care.
